# Cutaneous onchocerciasis in Dumbu, a pastoral area in the North-West region of Cameroon: diagnostic challenge and socio-economic implications

**DOI:** 10.11604/pamj.2015.22.298.7707

**Published:** 2015-11-24

**Authors:** Tsi Njim, Joel Mbigha Ngum, Leopold Ndemnge Aminde

**Affiliations:** 1 Regional Hospital Bamenda, Bamenda, Cameroon; 2 District Hospital Fundong, Fundong, Cameroon; 3 Sub-divisional Hospital Nguti, Nguti, Cameroon; 4 Clinical Research Education, Networking and Consultancy (CRENC), Douala, Cameroon

**Keywords:** Onchocerciasis, neglected tropical disease, blindness, Dumbu, Cameroon

## Abstract

Onchocerciasis is a severe parasitic infestation caused by *Onchocerca volvulus* which causes disabling skin and subcutaneous tissue changes and ultimately leads to blindness. It has a huge public health impact due to its socioeconomic burden and the vast number of people it affects in developing countries. In this case, a 60 years old woman was encountered with leopard skin like changes, rashes and pruritus on the left leg; which had been managed as cutaneous mycosis for over a period of 8 years. A diagnosis of onchocerciasis was finally made after a skin snip identified onchocercal microfilariae. The above case shows that onchocerciasis is still a neglected tropical disease (NTD) in Cameroon. This emphasizes the need for more expansive outreach programs in remote areas in Cameroon, a change in health policies to ensure the eradication of this disabling disease and health promotion amongst vulnerable populations.

## Introduction

Onchocerciasis is a severe disabling disease which affects the skin and subcutaneous tissues of humans [1]. This parasitic infestation caused by *Onchocerca volvulus* is transmitted to humans through the bite of a black fly (genus Simulium) [2]. From causing unpleasant skin symptoms, the disease progresses to cause intense inflammatory reactions in soft tissues. In the eye, the final consequence of this reaction is blindness [1]. The public health significance of this disease cannot be overemphasized; with an estimated 17 million people infected worldwide and over 270,000 people blinded by the disease [1, 3–6]. The situation in Africa is worrisome with an estimated 99% of all cases of onchocerciasis and onchocerciasis-related blindness reported to be from this continent [4]. In Cameroon, over 60% of the rural population is at risk of infection with Onchocerciasis [4]. We report a case of cutaneous onchocerciasis in Dumbu, a pastoral and remote village in the North west region of Cameroon; with limited access to health care services.

## Patient and observation

**Figure 1:**
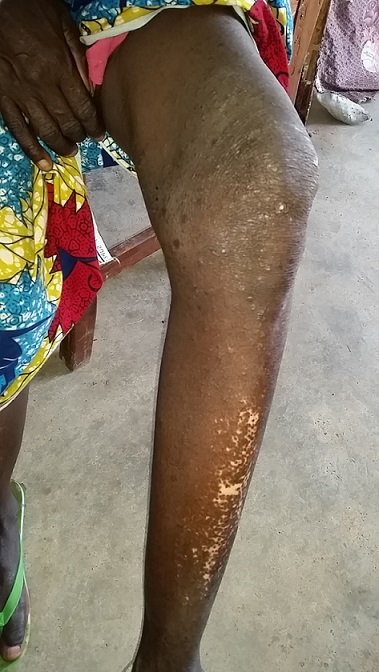
Left leg of patient showing hypopigmented changes (leopard skin appearance) to the shin and papular lesions around the knee

A 60 year old woman encountered during an outreach consultation, presented with skin changes and a rash on the left leg associated with severe pruritus over a period of 8 years. She consulted with the local nurse who made a provisional diagnosis of a cutaneous mycosis; and treated her with Griseofulvin for over 4 years. Her house was situated by a slow-flowing stream. On examination, there was a papular rash around the left knee associated with excoriation marks probably due to scratching. There was hypopigmentation around the shin of her left leg (leopard skin-like changes) with associated hardening of the skin around this region (Figure 1). A skin snip of the left leg was obtained and prepared with normal saline. When viewed under a microscope after two hours, microfilariae were seen wriggling free on the slide. She was stopped from taking Griseofulvin; given 12mg of Ivermectin in a single dose and Loratadine. She returned 3 months later for a follow-up; with disappearance of the pruritic papular lesions.

## Discussion

From a single bite of a black fly, an individual could suffer from permanent disability; blindness. After a painful bite from the Simulium fly, infective larvae of *Onchocerca volvulus* are introduced into the skin. The larvae mature into adult worms in 2 to 4 months and can live in subcutaneous tissue for several years [5]. Fibrosis around the adult worm causes characteristic nodules around bony prominences [5, 7]. These adult worms produce several microfilariae which migrate in subcutaneous tissues; the live microfilariae cause minute tissue reactions while the dead microfilariae elicit a severe tissue allergic inflammatory reaction which could lead to necrosis. In the skin, this reaction leads to a pruritic papular rash, scarring and a leopard skin appearance after patchy hypopigmentation in chronic cases [5]. In the eye, this reaction leads to sclerosing keratitis, pannus formation, uveitis, conjunctivitis and iridocyclitis [4]; which ultimately lead to blindness. The burden of infection with onchocerciasis spans through affecting not only individuals but communities as well. Significant morbidity has been reported among individuals affected by the disease [5, 8]. From skin changes with its associated pruritus to river blindness, an individual infected with this disease is limited from several social and economic activities [9]. This leads to dependence on other community members and hence a vicious cycle of poverty and disease. Moreover, in our case, the patient was committed to inappropriate long-standing treatment with Griseofulvin due to a misdiagnosis; leading to unnecessary expenditure and possibly exposure to eventual side effects of chronic Griseofulvin consumption. Onchocerciasis may also not be directly related to mortality, but is known to decrease the host immunity of the infected individual with consequent reduction in life expectancy by up to 13 years [10]. Communities have been known to be socioeconomically suppressed by this NTD. Large fertile areas located along fast-flowing rivers have been evacuated and left uncultivated due to fear of the disease [10]. This represents a great socioeconomic difficulty especially to rural populations who depend mostly of farming and cattle rearing as a source of revenue. Furthermore, it has been postulated that if the health burden of onchocerciasis is not decreased considerably by governments, several developing countries will face severe difficulties attaining the millennium development goals (MDGs) [10].

Thus, onchocerciasis although neglected, is unquestionably a disease with significant morbidity and mortality. It therefore has a high public health impact and countries should strive towards the elimination of the disease. Several countries have already been successful in this endeavor with Colombia being the first country to totally achieve eradication of transmission of the disease in 2013. Several Latin American countries have followed suit with Guatemala, Mexico, Ecuador and Brazil requesting verification of elimination of transmission of the disease from international bodies [3]. These countries have succeeded in controlling this disease through the use of the highly successful vector control program (OCP-Onchocerciasis Control Program) and the use of annual treatment using Mectizan (MDP-Mectizan Distribution Program). The combination of these two programs known as the Onchocerciasis Elimination Program in the Americas (OEPA) has shown an exceptional achievement towards realization of eradication in the Americas [3]. However, Cameroon amongst other African countries is lagging behind. Though a program aimed at eradication; the African Program for Onchocerciasis Control (APOC) modelled after the highly successful OEPA exists [3, 10], several African countries still struggle with this disease. In Cameroon for example, health care providers (HCP) capable of identifying zones of high risk are not available. The people of Dumbu do not have a HCP who can adequately diagnose Onchocerciasis. The lady in the above case presentation had suffered from disabling conditions of the disease for over 8 years, prior to a definitive diagnosis made under fortuitous circumstances. Furthermore, the distribution of annual treatment (Mectizan) is highly inadequate as most individuals in such remote areas do not receive the treatment due to the inaccessibility of such regions; most being probable endemic areas of Onchocerciasis. Finally, there's the complete absence of vector control programs which if coupled with annual distribution of treatment may go a long way in controlling the disease. The above observations bring to light gaps in public health policies and provides evidence for the nil success of APOC in Cameroon.

### Recommendations

Firstly, training of HCP working in remote areas on the recognition and management of Onchocerciasis would be very beneficial in control and subsequent eradication of the disease. Secondly, poor accessibility has most likely led to poor coverage and persistence of the disease. The creation of better roads to access remote areas should be considered to make these areas (most of which are high risk zones) accessible to HCP during distribution of annual treatment for Onchocerciasis. Thirdly, massive health promotion campaigns should be encouraged in remote areas like Dumbu in Cameroon. Communities should be empowered with knowledge especially on prevention of the disease, some of which may include; wearing long dresses especially in the evenings, keeping their surroundings free from bushes, avoiding swampy areas and relocating from areas close to fast flowing streams. Finally, vector control was an important factor in the success of OEPA in the Latin Americas. It is absolutely necessary that this program be attempted in Cameroon for the control of Onchocerciasis.

## Conclusion

Onchocerciasis is still very much a NTD in Cameroon. This is probably due to gaps in public health policies instituted to control this disease. Individuals, especially those in rural and endemic areas are thus victims of the disabling conditions associated with this disease with consequent repercussions on the socioeconomic development of the country. Interventions for the control of this disease need to be modified and or intensified to include vector control, regular and effective annual distribution of Mectizan, community health education and adequate training of HCP towards recognizing and managing the disease.
